# The Effect of DNA-Dispersed Single-Walled Carbon Nanotubes on the Polymerase Chain Reaction

**DOI:** 10.1371/journal.pone.0094117

**Published:** 2014-04-04

**Authors:** Ryan M. Williams, Sara Nayeem, Bridget D. Dolash, Letha J. Sooter

**Affiliations:** Basic Pharmaceutical Sciences, West Virginia University, Morgantown, West Virginia, United States of America; UT MD Anderson Cancer Center, United States of America

## Abstract

The unique properties of single-wall carbon nanotubes (SWCNT) make them useful in many new technologies and applications. The interaction of DNA and SWCNT is of interest for many uses, including molecular sensors. This study determined polymerase chain reaction (PCR) efficiency in amplifying a 76 base pair DNA sequence in the presence of SWCNT, of heterogeneous “Mix” and (6,5)-enriched chiralities, associated with three DNA sequences. The dependence of PCR efficiency on the concentration of DNA:SWCNT preparations was measured, as well as their age and level of dispersion (less than one month or between four and ten months). Additionally, the ability to directly amplify the DNA sequence associated with the SWCNT scaffold was investigated. In PCRs with DNA:SWCNT preparations less than one month old, concentrations greater than or equal to 0.1 mg/mL inhibited the PCR reaction. In PCRs with older preparations, no inhibition was seen at 0.01 or 0.1 mg/mL, with amplification at 1 mg/mL in some samples. Additionally, our studies showed that the DNA directly associated with the SWCNT can be amplified using PCR. This work provides an inhibitory concentration of DNA-dispersed SWCNT in PCR reactions for different preparations as well as a basis for future DNA:SWCNT studies that require PCR amplification. This will be useful for future studies focused on the use of SWCNT in molecular sensing technologies.

## Introduction

Single-wall carbon nanotubes (SWCNT) are cylindrical fullerenes with walls of single carbon atom thickness [1]–[3]. They have enormous potential in industrial, sensing, and biomedical technologies due to their unique physical, electronic, and optical properties [4]–[7]. SWCNT may be either metallic or semiconducting, with 33% and 67%, respectively, of all potential species having those characteristics [8], [9]. The unique properties of SWCNT are dependent upon the chirality and diameter of the SWCNT, however purification of individual species remains difficult [9]. Individual types of semiconducting SWCNT have unique optical fluorescence properties [10]. Therefore, in applying SWCNT, it is possible to take advantage of their electrical or optical properties, separately or combined [11].

Single-stranded DNA has been used to disperse hydrophobic SWCNT in solution [12]. The interaction of DNA sequences with SWCNT is useful in molecular sensing and purification techniques. Individual sequences have been identified that preferentially wrap certain SWCNT chiralities and will be useful in purification of individual chiral species [13]–[16]. DNA-associated SWCNT have also been used in molecular sensing, and it is likely that amplification of the DNA sensing element may be necessary in the presence of SWCNT [17]–[19]. The potential use of a DNA-wrapped SWCNT (DNA:SWCNT) platform shows promise in multiple applications; however research is continuing to optimize use of these complexes.

In sensing applications and analysis of environmental samples, it is useful to employ polymerase chain reaction (PCR) techniques in order to exponentially amplify the SWCNT-associated DNA sequence. Additionally, there has been work to develop PCR-based microfluidic sensing devices [20], as well as DNA-functionalized SWCNT sensing devices [19]. Integration of these two sets of technologies would create new potential analyte detection mechanisms. Therefore, it is important to determine the effect of DNA-associated SWCNT on the polymerase chain reaction.

The aim of this study was to investigate amplification of DNA by the polymerase chain reaction (PCR) in the presence of DNA-dispersed SWCNT. We have chosen a DNA sequence broadly representative of those that will be amplified in the presence of SWCNT: that of an estradiol (E2) Molecular Recognition Element (MRE) with 57% GC content [21]. Factors investigated include DNA sequence-dependence, SWCNT chirality-dependence, and DNA:SWCNT concentration-dependence; amplification of the wrapping sequence itself; and age of the DNA-SWCNT preparation. This work will provide a basis for further DNA-dispersed SWCNT studies which require PCR amplification with a focus toward molecular sensor development.

## Materials and Methods

### DNA-dispersed SWCNT preparation

DNA:SWCNT preparations were made similarly to previously described methods [12], [22]. Briefly, 2 mg CoMoCat SWCNT were mixed with 2 mg DNA in 2 mL phosphate-buffered saline (PBS). SWCNT used were: without chiral enrichment “Mix” or (6,5) chirality-enriched (Sigma-Alrich; St. Louis, MO). DNA sequences used were: E2 MRE [21], TG15 (Table 1) (Eurofins MWG Operon; Huntsville, AL), or salmon testes genomic DNA (Sigma-Aldrich; St. Louis, MO). This mixture was sonicated on ice for two hours with a Virsonic XL2020 ultrasonic liquid processor (Misonix; Farmingdale, NY) equipped with a 3.2 mm microtip at approximately 20% power. The resulting solution was centrifuged at 16,000×g for 90 minutes to remove insoluble SWCNT. The solution was then filtered through an Amicon Ultra-4 Centifugal Filter unit with 100,000 molecular weight filter (Millipore; Billerica, MA) three times at 4000 RPM for 15 minutes each to remove DNA that did not associate with the SWCNTs. The recovered DNA:SWCNT solution was brought to a volume of 500 μL with PBS. The concentration of the sample was obtained by gravimetric analysis of 10 μL of the solution weighed on an Ohaus Discovery Microbalance (Ohaus; Parsippany, NJ). Samples were stored in screw-top containers at room temperature before use. To determine the effect of DNA:SWCNT preparation age on PCR, samples were used that were prepared between four and ten months before assayed or less than one month before assayed. In total, 12 DNA:SWCNT samples were prepared.

**Table 1:** Synthesized DNA sequences used.

E2 MRE	5′-GCTTCCAGCTTATTGAATTACACGCAGAGGGTAGCGGCTCTGCGCA TTCAATTGCTGCGCGCTGAAGCGCGGAAGC-3′
TG15	5′-TGTGTGTGTGTGTGTGTGTGTGTGTGTGTG-3′
F.E2	5′-GCTTCCAGCTTATTGAATTACACGCAGAGGGTAGC-3′
R.E2	5′-GCTTCCGCGCTTCAGCGCGC-3′

### SWCNT characterization

Absorption and near-infrared (NIR) fluorescence measurements were performed as previously described with few modifications [23]. Briefly, each DNA:SWCNT preparation was diluted to a 1% solution with distilled-deionized water (Millipore; Billerica, MA). This solution was added to a 10 mm path length cuvette (Starna Cells; Atascadero, CA) and analyzed on a NanoSpectralyzer 1 (NS1) (Applied NanoFluorescence; Houston, TX). The absorbance spectrum was collected from 400–1600 nm. Additionally, fluorescent emission spectra were measured using excitation wavelengths of 638 nm, 690 nm, and 784 nm. The resulting spectra were analyzed with ANFSoft (Applied NanoFluorescence; Houston, TX). Fluorescence efficiency measurements, which are fluorescence intensity (RFU) collected across the emission spectrum for a single wavelength divided by the absorbance spectrum, are reported as a relative measure of SWCNT dispersion in solution.

### Polymerase chain reaction in the presence of DNA-dispersed SWCNT

In total, 16 sets of PCR experiments were performed involving 7 individual PCR reactions per set. Standard conditions for PCR amplification of the E2 MRE were utilized similar to as previously described [21], [24], [25]. These were: 60 nM E2 MRE, 400 nM forward and reverse E2 MRE primers (F.E2 and R.E2) (table 1), 250 μM deoxyribonucleotide triphosphates, 1X GoTaq Reaction Buffer (Promega; Madison, WI), 3.5 units *Taq* DNA Polymerase, DNA:SWCNT suspension, and Milli-Q water to 50 μL. DNA:SWCNT were added to final concentrations of: 0.01 mg/mL, 0.1 mg/mL, 1 mg/mL, 5 mg/mL, 10 mg/mL. For each set, a negative control was performed with no E2 MRE free PCR template or DNA:SWCNT and a positive control was performed with only free PCR template and no DNA:SWCNT added. Reactions were performed on an MJ Mini Thermal Cycler (Bio-Rad; Hercules, CA) with conditions: initial denaturation of 95°C for 5 minutes; 20 cycles of 95°C for 1 minute 30 seconds, 68°C for 45 seconds, and 72°C for 1 minute 30 seconds; and final extension of 72°C for 7 minutes. Amplification of DNA directly associated with SWCNT was performed using E2 MRE:SWCNT preparation with no additional E2 MRE added to the reaction mix, testing the ability to amplify DNA associated with SWCNT. Products of control and DNA:SWCNT PCR reactions were run on a 4% agarose gel containing 5 μg/mL ethidium bromide for 30 minutes at 135 volts in Tris:Borate:EDTA (TBE) buffer. Five μL of each PCR and 3 μL of 100 bp and 25 bp ladder (Promega; Madison, WI), were mixed with 2 μL 6X loading dye (Promega; Madison, WI) prior to loading in the well. Gels imaged with UV illumination on a BioRad Gel Doc System and analyzed on Quantity One Software (Bio-Rad; Hercules, CA).

## Results and Discussion

### Analysis of DNA:SWCNT preparations

**Figure 1:**
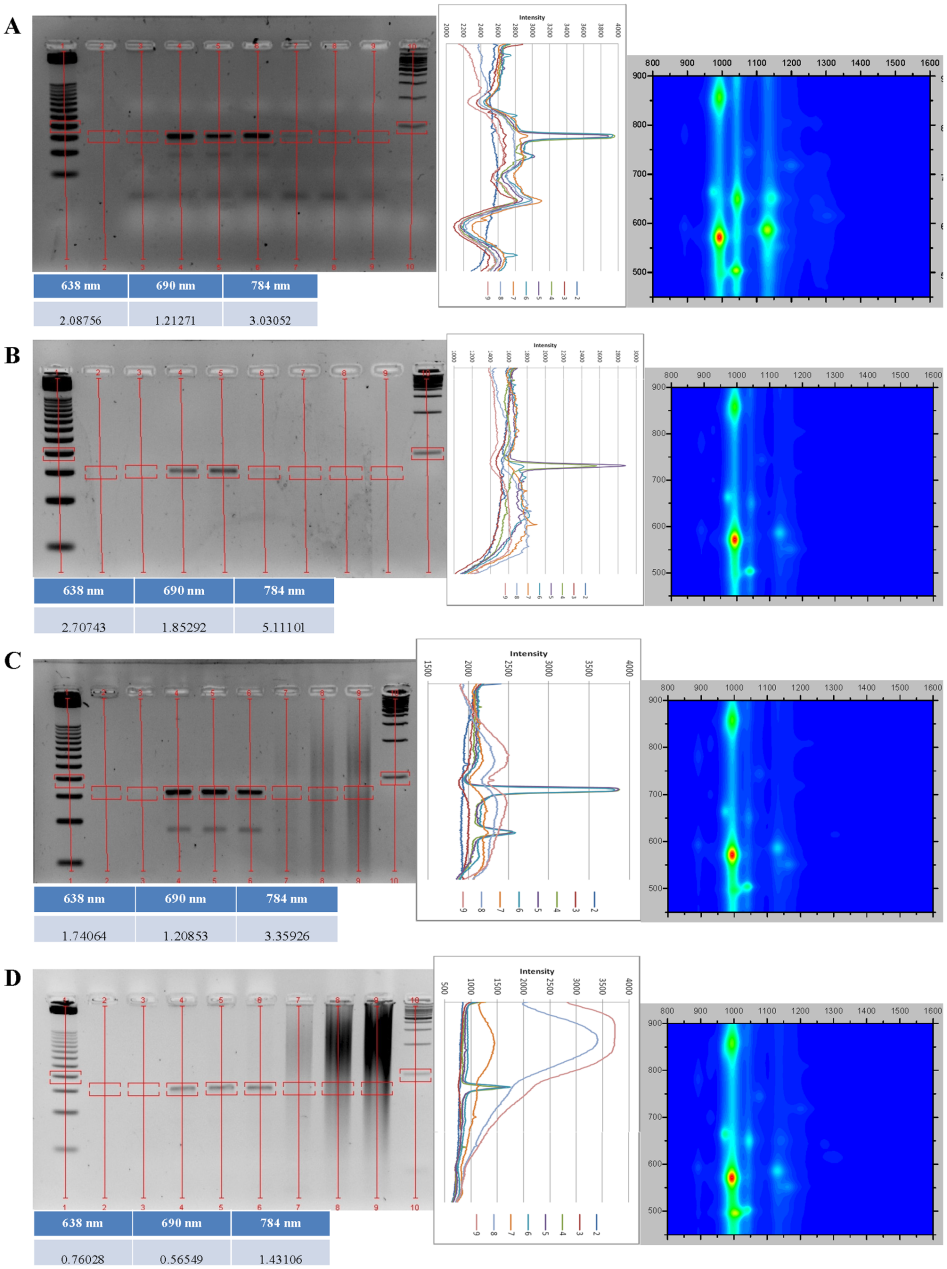
Agarose gel electrophoresis images and analysis of gel images and old (4–10 months) DNA:SWCNT samples, set one. For each panel, top left is agarose gel image with lanes and quantitized boxes shown, top center is intensity line graph, top right is NS1 synthesized fluorescence intensity plot of DNA:SWCNT samples (y-axis is excitation wavelength, x-axis is emission wavelength), bottom left is fluorescence efficiency at each excitation wavelength in NS1 analysis (higher numbers correlate with greater dispersion). Lanes in agarose gel images and intensity line graph: 1. 25 bp ladder (black) 2. unloaded empty well (dark blue) 3. negative control with no SWCNT or PCR template (red) 4. positive control with only PCR template and no SWCNT (green) 5. 0.01 mg/mL DNA:SWCNT (purple) 6. 0.1 mg/mL (neon blue) 7. 1 mg/mL (orange) 8. 5 mg/mL (light blue) 9. 10 mg/mL (pink)10. 100 bp ladder (black). a) TG15:Mix; b) TG15:(6,5); c) Salmon:Mix; d)Salmon:(6,5). (By convention, naming of DNA:SWCNT complexes is as follows: “DNA sequence: type of SWCNT”.)

**Figure 2:**
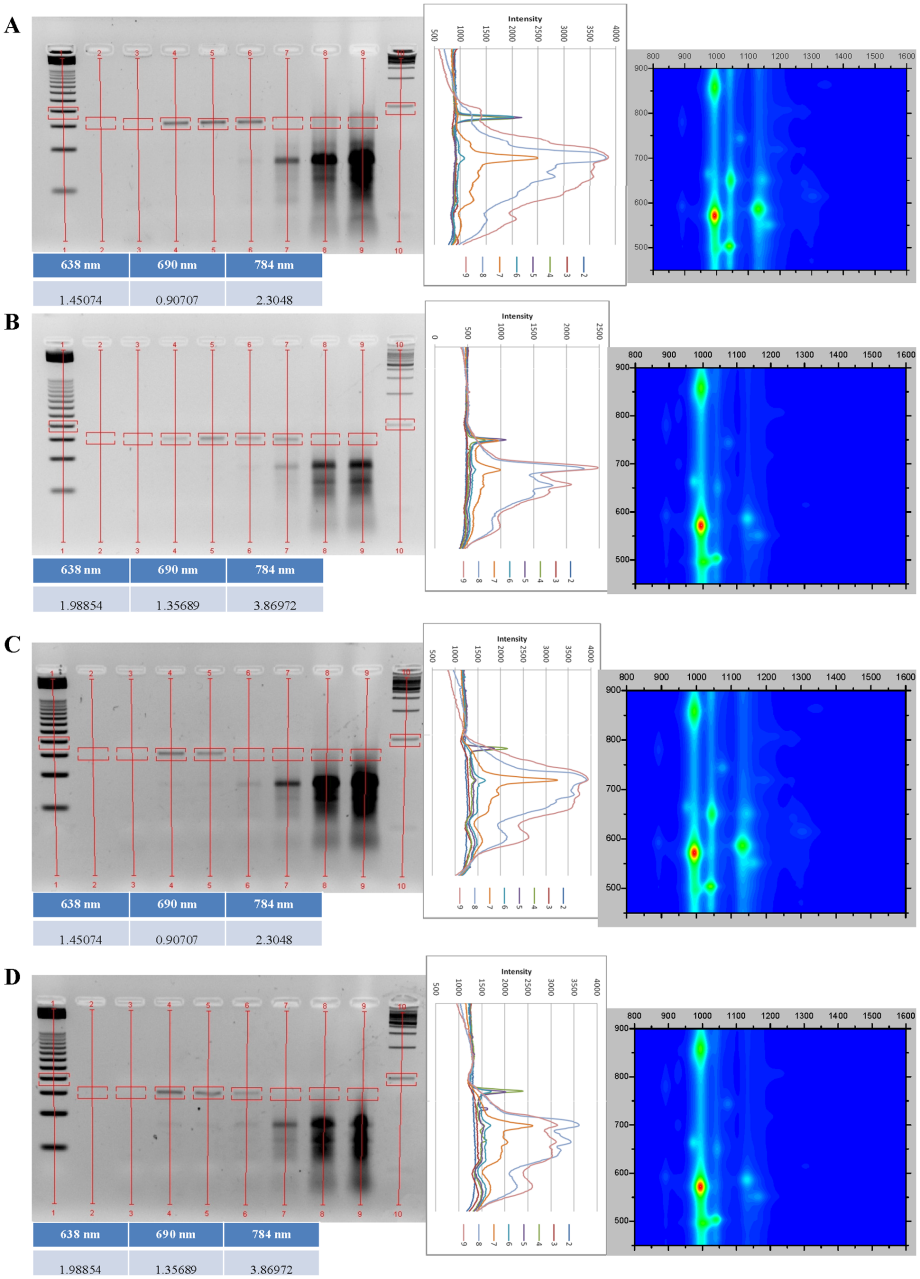
Agarose gel electrophoresis images and analysis of gel images and old (4–10 months) DNA:SWCNT samples, set two. All parts of each figure panel are the same as in Figure 1. a) E2 MRE:Mix; b) E2 MRE:(6,5); c) E2 MRE:Mix, No free template MRE; d) E2 MRE:(6,5), No free template MRE.

**Figure 3:**
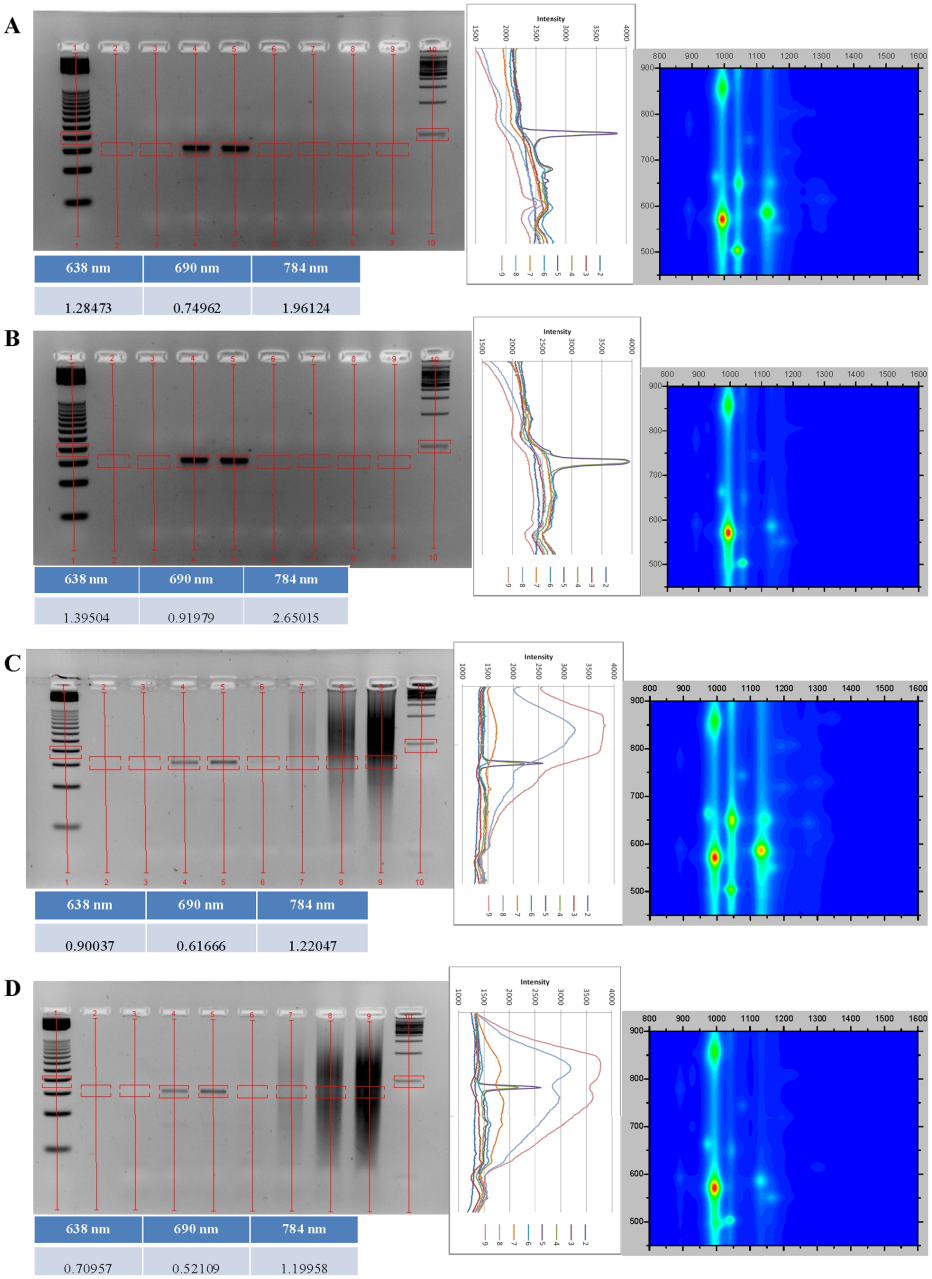
Agarose gel electrophoresis images and analysis of gel images and new (<1 month) DNA:SWCNT samples, set one. All figure notes are the same as in Figure 1.

**Figure 4:**
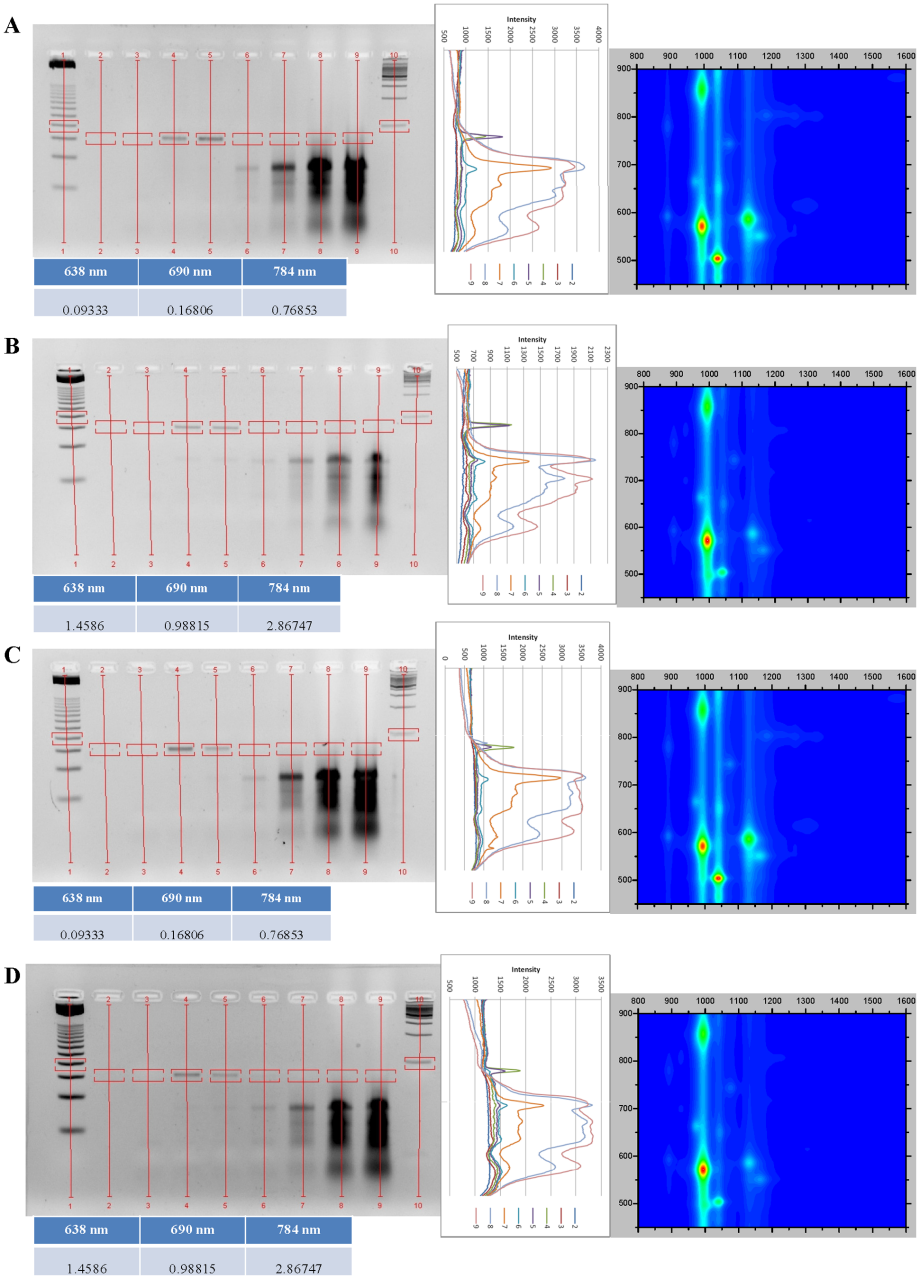
Agarose gel electrophoresis images and analysis of gel images and new (<1 month) DNA:SWCNT samples, set two. All figure notes are the same as in Figure 2.

All DNA:SWCNT samples were analyzed by absorbance and NIR fluorescence spectroscopy to determine their SWCNT chirality composition and dispersion in solution (singly-dispersed SWCNTs) (Fig. 1, 2, 3, 4). DNA:SWCNT samples greater than four months old were more well-dispersed than the identical DNA:SWCNT preparation of less than one month. This would suggest that dispersion of SWCNT in solution with DNA increases with time. Additionally, for both old and new samples, TG15 and E2 MRE dispersed the (6,5)-enriched SWCNT better than mixed chirality SWCNT (Fig. 1, 2, 3, 4, fluorescence efficiency). These results are consistent with previously published results that have shown short, synthetic DNA sequences rich in thymine and guanine have a high affinity for (6,5) chiral SWCNTs [26]. Conversely, the salmon testes genomic DNA:Mix SWCNT samples were more well-dispersed than Salmon testes genomic DNA:(6,5) SWCNT. These results suggest that although the genomic DNA shows the highest association with (6,5)-chiral SWCNTs (Fig. 1, 2, 3, 4, topography plots) it also forms stable associations with other chiralities.

### Agarose gel analysis of old DNA:SWCNT PCRs

Agarose gel analysis of PCR amplification of the positive control reaction with only free PCR template and no SWCNT confirm optimized amplification conditions. PCRs performed with increasing concentrations of DNA:SWCNT “old” preparations suggest that accurate amplification products of 76 base pairs (bp) can be obtained at concentrations of 0.1 mg/mL and below for all preparations (Fig. 1 & 2, agarose gels). As an overall trend, PCR amplification is either unaffected or slightly increased in the presence of 0.01 and 0.1 mg/mL DNA:SWCNT compared to the positive control reaction with free PCR template and no DNA:SWCNT added (Fig. 1 & 2, intensity graphs). The exception to this is the TG15:(6,5) sample (Fig. 1B), where a very faint band is produced at 0.1 mg/mL. Additionally, bands are produced at 1 mg/mL for TG15:Mix and E2:(6,5) PCRs. No distinct bands are produced at 5 or 10 mg/mL concentration PCRs for any preparation. Prior work has shown that SWCNT sonicated in water just before being added to a PCR reaction increased amplification of a 410 bp product at concentrations up to 3 mg/mL [27]. The same study showed that SWCNT in a PCR reaction interacts with both the DNA template as well as *Taq* DNA Polymerase. This study, however, has DNA associated with the SWCNTs, so this is may not be the source of inhibition due to strong association of DNA and SWCNT. Additionally, the slight increase in PCR efficiency with the lowest levels of PCR:SWCNT suggests *Taq* interaction with SWCNT is not inhibitory at lower concentrations. A critical concentration of nanotubes likely exists which inhibits reaction components from interacting or increases *Taq* interaction. Another study showed that that SWCNT functionalized with carboxylic groups produce stronger interactions with *Taq* and more inhibition of PCR than did pristine SWCNT at 0.1–0.8 mg/mL [28]. Therefore, it is possible there is some adsorption of *Taq* DNA Polymerase onto DNA:SWCNT, but it is unlikely to be as strong of an interaction and inhibitory effect than with carboxylic SWCNT.

It is also possible to use PCR to amplify the E2 MRE target which is only present in the reaction associated with SWCNTs (Fig. 2C, D). These concentration-dependent patterns follow a similar pattern to reactions performed with the addition of free E2 MRE. This is important in that it shows the ability to directly amplify DNA which is associated with SWCNT.

It is also clear that addition of DNA:SWCNT to PCR reactions has effects on amplicons produced. In both Salmon:SWCNT samples, smears are visible in lanes which there is no distinct E2 MRE band (Fig. 1C, D, intensity graphs). This is likely due to degradation of the genomic DNA present in the sample and non-specific amplification. In both E2:SWCNT samples amplification of a product just less than 50 bp increases with E2:SWCNT concentration (Fig. 2A, B). This is also true for E2:SWCNT PCRs performed with no free E2 MRE in the reaction. This is likely due to an excess of the amplification target itself in the reaction. However, in the TG15:SWCNT PCRs, no amplicons appeared that were not present in the control reaction.

### Agarose gel analysis of new DNA:SWCNT PCRs

Analysis of PCR experiments performed with DNA:SWCNT samples prepared less than a month before assayed show amplification in the presence of 0.01 mg/mL (Fig. 3 & 4, agarose gels). At this concentration, all PCR experiments performed in the presence of free E2 MRE showed an increase in amplification (Fig. 3 & 4, intensity graphs). However, in contrast to PCR experiments with “old” DNA:SWCNT samples, no amplification of E2 MRE was seen at or above concentrations of 0.1 mg/mL. This result suggests that more dispersed SWCNT, as seen in the older samples, is less inhibitory to PCR reactions. This may be because more agglomerated SWCNT hinder the reaction components from encountering each other. This is supported by the fact that higher concentrations have an inhibitory effect on PCR efficiency. However, in both old and new samples, there seems to be no difference in PCR efficiency between Mix or (6,5)-enriched SWCNT samples. This is in spite of a difference in dispersion based on DNA sequence.

In PCR experiments in the presence of “new” E2:SWCNT samples and absent of free E2 MRE, the MRE was amplified from the SWCNT scaffold (Fig. 4C, D). Amplification of the 76 bp product is observed in the presence of 0.01 mg/mL E2:SWCNT, however not at higher concentrations. This follows the same pattern seen as PCR reactions done in the presence of E2:SWCNT and the free E2 MRE. There is, however, a reduction in efficiency at this concentration, which contradicts amplification of free E2 MRE at this concentration.

Amplicons produced from PCR reactions in the presence of new DNA:SWCNT followed the same general pattern as those with old samples. Previous studies have shown SWCNT increase the specificity of long PCR (14 kb) up to the point that the reaction is inhibited [29]. The difference in results may be a function of size of the reaction product, or may be due to the method of SWCNT dispersion, which was sonication in water. Another study has shown that different concentrations of carbon nanopowder (CNP) does not affect amplicons produced in a PCR reaction [30]. Therefore, the effects are likely due to degradation of salmon genomic DNA or excess target present in the reaction as previously discussed.

Future work will determine the effects of DNA-dispersed SWCNT on PCR amplification of longer templates as well as those with varying base composition. Additionally, while it is clear that DNA:SWCNT inhibit PCR amplification, the mechanism of this will be determined in further extension of this work.

## Conclusions

This work shows differential inhibitory concentrations for old and new DNA:SWCNT preparations of varying levels of dispersion in PCR reactions at concentrations of 0.1 and 0.01 mg/mL, respectively. The inhibitory mechanism and effect on different PCR templates will be fruitful to investigate in future work. We also demonstrate that it is possible to amplify a DNA sequence that is directly associated with SWCNTs through sonication-mediated dispersion. This is important for both DNA:SWCNT-based molecular sensors and environmental sample testing applications and lays the groundwork for future studies focused on these technologies.
